# An Applied Research Partnership to Improve The Children’s Behavioral Health System Of Care

**DOI:** 10.23889/ijpds.v9i5.2587

**Published:** 2024-09-18

**Authors:** Ted McDonald, Pablo Miah

**Affiliations:** 1 University of New Brunswick

## Objective

As in many jurisdictions, New Brunswick (Canada) is facing increasing shortages of K-12 school teachers as retirements loom at the same time that the school age population of NB continues to exceed long term trends. The purpose of this retrospective study is to analyze the recruitment, retention and exit decisions of teachers in the NB public education system in order to support ongoing planning around teacher staffing.

## Approach

The analysis uses a unique linked administrative data combining province-wide individual-level teacher employment data, immigration records, university graduation data and public health insurance registration on NB teachers and individuals who obtained a B.Ed. degree in NB. Data are accessed through the secure facilities of the NB Institute for Research, Data and Training. The analysis includes both descriptive statistics and econometric methods appropriate to the particular outcomes of interest.

## Results

The analysis will present results on three types of labour market transitions: 1) from NB university education to employment as a NB teacher. 2) exits from employment as a teacher, including both retirement and pre-retirement exits, and 3) decisions of ex-teachers about remaining in NB. The potential effects of a range of demographic, geographic and system-level factors on these outcomes are considered.

## Conclusions and Implication

Results from the analysis of entry to and exit from K-12 teaching in NB will be vital to human resource planning for a province dealing with looming retirements, growing labour shortages and an increasing population. Future work will consider subject-specific teacher shortages in critical fields like STEM.

